# HLA-G14bp ins/del polymorphism and post-transplant weight gain in kidney transplantation: potential implications beyond tolerance

**DOI:** 10.1186/s12882-020-01752-6

**Published:** 2020-03-30

**Authors:** Daniela Piancatelli, Daniela Maccarone, Alessia Colanardi, Pierluigi Sebastiani, Katia Clemente, Samuele Iesari, Quirino Lai, Francesco Pisani

**Affiliations:** 1 National Research Council (CNR) - Institute of Translational Pharmacology (IFT), Via Carducci, 32, 67100 L’Aquila, Italy; 2 Regional Center for Organ Transplantation (CRT), S. Salvatore Hospital, L’Aquila, Italy; 3 General Surgery and Organ Transplantation, S. Salvatore Hospital, L’Aquila, Italy; 4 Pôle de chirurgie expérimentale et transplantation, Institut de recherche expérimentale et clinique, Université catholique de Louvain, Brussels, Belgium; 5 Department of Biotechnological and Applied Clinical Sciences, University of L’Aquila, L’Aquila, Italy; 6 Transplant Unit, University “La Sapienza”, Rome, Italy

**Keywords:** HLA-G, Gene polymorphism, Kidney transplant, Obesity, Immunogenetics

## Abstract

**Background:**

Human leukocyte antigen (HLA)-G is a non-classical HLA molecule with immunomodulant and immunosuppressive functions, involved in transplantation tolerance. HLA-G14bp ins/del polymorphism in exon 8 has been associated with allograft rejection and kidney transplant outcome, with controversial results. We investigated associations of HLA-G14bp ins/del polymorphism on onset of some of the main post-transplant risk factors, like excess body weight, lipid abnormalities, increased fasting plasma glucose. Polymorphisms of cytokines with both immunosuppressive and metabolic effects were also assessed for comparisons and associated analysis.

**Methods:**

The present study involved kidney transplant recipients (*n* = 173) in which body mass index, cholesterol, triglycerides, fasting plasma glucose were registered in the first years after transplantation and analyzed in association with genotypes. Presence of hypertension and smoking habits, demographic, transplant-related and therapeutic data of patients were also recorded. Polymerase chain reaction, sequence-specific primer amplification and Taqman allelic discrimination techniques were used for genotyping of HLA-G14bp ins/del, interleukin (IL)-10(−1082G > A,-819 T > C,–592A > C), transforming growth factor-β(+ 869 T > C,+915C > G), IL-6(−174G > C), tumor necrosis factor-α(−308G > A) and IL-18(−137G > C,-607C > A). Effects of genotypes on clinical markers at each time point (pre-transplant and 1 to 5 years after transplant) were analyzed using a repeated-measures general linear model analysis; adjustment for potential confounders was performed.

**Results:**

Results showed that HLA-G14bp ins/ins was significantly associated with obesity, in particular after transplantation (3 years, *p* = 0.002, OR = 4.48, 95% CI:1.76–11.41). Post-transplant body mass index was significantly increased in HLA-G14bp ins/ins carriers (3 and 4 years, *p* = 0.033 and *p* = 0.044); effects of HLA-G14bp genotypes on post-transplant BMI were confirmed by using repeated-measures analysis and after controlling for confounding variables. Cytokine genotypes did not associate with the examined factors.

**Conclusions:**

The study of transplanted patients allowed to evidence a potential relationship between post-transplant weight gain and HLA-G14bp ins/del polymorphism, previously involved in rejection for its immunosuppressive/tolerogenic activity. This novel association could widen the knowledge of the role and functions of HLA-G molecules in diseases and transplantation.

## Background

Immunogenetic polymorphisms of molecules involved in allograft rejection and tolerance could have an impact on pathogenic mechanisms, which promote or prevent risk for post-transplant cardiovascular and metabolic morbidity. It is the case of some cytokines, which were extensively investigated in both transplanted patients and cardiovascular and metabolic diseases.

Obesity, dyslipidemia, type 2 diabetes mellitus (T2DM) and arterial hypertension are known post-transplant risk factors for cardiovascular diseases and allograft dysfunction: they are involved in allograft survival, metabolic syndrome, and increased post-transplant cardiovascular complications and mortality [1].

Body mass index (BMI) is the most common measure of obesity. Increased BMI is now a common finding in transplant candidates, more than in the past. Increased BMI (in particular over 30 kg/m^2^) was recently confirmed as a predictor of both early (acute rejection and delayed graft function) and long-term adverse kidney transplant outcome in a large study [2]. Post-transplant weight gain affects both normal-weight and overweight/obese patients (about 9 − 14 kg in the first year after transplantation and from 11% in the first year to 15% over 5 years) [3].

Both cardiovascular, metabolic, infectious diseases and malignancies are promoted as a part of adverse effects of post-transplant immunosuppressive medications, together with pre-transplant and general risk factors. Interactions between immune and metabolic mechanisms are known [4]; relationships exist between obesity, inflammation and decreased immunological tolerance, as indicated by studies in allergic, infectious diseases and cancer [5–7].

In human conditions such as obesity, a low-grade inflammation with increased levels of pro-inflammatory markers is present; some inflammatory cytokines (i.e., interleukin (IL)-6, IL-18) are involved in BMI increase, metabolic and cardiovascular diseases [8–10] and anti-inflammatory interventions can, at least in part, counteract metabolic dysfunctions.

Both the delayed immune response directed towards the transplanted kidney and injuries directed towards different tissues in recipients are mediated by molecules that show a certain grade of genetic variability. Functional polymorphisms of genes encoding immunosuppressive and tolerogenic molecules could be potentially predictive of post-transplant risk. Human Leukocyte Antigen (HLA)-G is a non-classical HLA molecule with immunomodulant and immunosuppressive functions. The most common HLA-G polymorphism consists of an insertion/deletion (ins/del) of 14 base pairs (bp) in exon 8 that induces changes of messenger ribonucleic acid (mRNA) stability, and protein expression [11]. Transplantation is a condition that could modify HLA-G expression. In kidney transplantation, HLA-G expression could be protective, because it inhibits alloreactive response towards the graft [12]. However, associations between HLA-G14bp genotypes and rejection were investigated, with inconclusive results [13–15]: an association of HLA-G14bp ins/ins genotype with acute rejection was found, although in a limited number of patients [13] and recipients with acute rejection were found to have lower soluble HLA-G (sHLA-G) levels [15]. Instead, HLA-G mRNA in graft biopsies was significantly higher in acute rejection, in comparison with patients with no rejection [16].

HLA-G expression can be modulated by other cytokines involved in proinflammatory, profibrotic and alloreactive responses. An example is represented by IL-10, which shares the anti-inflammatory properties and mechanisms of induction of tolerance with HLA-G. A previous study suggested an IL-10/HLA-G autocrine loop in physiological conditions, as an increase of IL-10 production from lipopolysaccharides (LPS)-activated peripheral blood mononuclear cells (PBMC) was preceded by sHLA-G release [17]. Recently, a role of HLA-G14bp polymorphism in vascular health has been suggested in kidney trasplant recipients [18].

Transplant recipients, generally being well-monitored and compliant patients during the first post-transplant years, represent a model for studying therapy-induced variations of risk factors and their interaction with some gene variants.

Based on these observations, our attention was focused on HLA-G14bp ins/del and other polymorphisms of cytokines involved in tolerance, inflammation and/or metabolic regulation (IL-10, Transforming Growth Factor (TGF)-β, IL-6, Tumor Necrosis Factor (TNF)-α, and IL-18); effects of genotypes on post-transplant weight gain and alterations of other risk factors for post-transplant complications were evaluated in kidney transplant recipients.

## Methods

### Patients

DNA samples were obtained from 173 Italian kidney transplanted patients from deceased donors (117 men and 56 women, mainly coming from Central Italy), regularly undergoing post-transplant follow up in the Unit of General Surgery and Transplantation of S. Salvatore Hospital of L’Aquila, Italy, where they were transplanted (between 2001 to 2010, except 2 patients transplanted elsewhere). The minimum follow-up was 36 months. Exclusion criteria were non-Caucasian race, age less than 18 years, pregnancy, severe diseases (malignancies or other diseases with poor prognosis in the short term, sepsis), primary non-function, graft loss within the first 3 years, irregular post-transplant monitoring or performed elsewhere, re-transplantation and transplantation from living donors.

Clinical features of patients and recorded parameters are reported in Table 1.

**Table 1:** Demographic and clinical characteristics of kidney transplant recipients (n = 173)

**Gender** (n, %)	M: 117 (67.6%) F: 56 (32.3%)
**Age at transplant** (years, mean ± SD)	46.77 ± 10.60 (range 24–68)
**Dialysis mode** (%)	19% peritoneal, 81% hemodialysis
**Time on dialysis** (years, mean ± SD)	2.81 ± 2.97 (range < 1–26)
**Immunosuppressive therapy:**	
• Calcineurin inhibitors (CsA or TAC), steroids, purine synthesis inhibitors (%)	84%
• mTOR inhibitors, steroids, purine synthesis inhibitors (%)	16%
**Pre-transplant impaired/diabetic fasting blood glucose**^a^(%)	13%
**Cold ischemia time** (hours, mean ± SD)	11.45 ± 3.31 (range 4.2–21.6)
**Recipients with acute rejection episodes** (n, %)	24 (13.9%)
**Graft loss**^b^(n, %)	4 (2.3%)
**Primary nephropathy**	
Glomerulonephritis^c^	73 (42%)
Polycystic kidney disease	33 (19%)
Nephroangiosclerosis	6 (4%)
Hypertensive/vascular nephropathies	19 (11%)
Chronic pyelonephritis	8 (5%)
Not determined	16 (9%)
Other nephropathies^d^	18 (10%)

^a^fasting plasma glucose: impaired: 111–125 mg/dl; diabetic: > 125 mg/dl (WHO)

^b^four cases of graft loss during the 5-years follow up (over 3 years post-transplant)

^c^chronic, membranous, focal glomerulosclerosis, rapidly progressive, LES, Goodpasture, IRC post-partum, diabetic, Alport syndrome, IgA nephropathy

^d^congenital, amyloid, neoplastic, post-traumatic, obstructive, hemolytic-uremic syndrome, contracted kidney, interstitial nephropathy

In addition, presence of hypertension (≥140/90 mmHg or use of antihypertensive drugs), the onset of T2DM, dyslipidemia (cholesterol> 200 mg/dl and/or triglycerides > 150 mg/dl, use of antidyslipidemic drugs), other pharmacological treatments (in addition to immunosuppressive therapy) and smoking habits were recorded.

All patients received induction therapy with basiliximab (anti-CD25 monoclonal antibody) and corticosteroids. Standard maintenance immunosuppressive regimens consisted of 1) calcineurin inhibitors (cyclosporine or tacrolimus), corticosteroids and inhibitors of purine synthesis (mycophenolate-mofetil or mycophenolic acid) (about 84% of patients), or 2) cyclosporine, corticosteroids and mammalian target of rapamycin inhibitors (mTORi, everolimus) (16%). Written informed consent was obtained from all subjects, investigations were carried out by the principles of the Declaration of Helsinki, and the study was approved by the local ethics committee (study n. 50/2008).

### Biochemical parameters

BMI, cholesterol, triglycerides, fasting plasma glucose, and creatinine were recorded before (i.e., baseline, at the time of the last half-yearly follow-up before the transplant) and after transplantation (each year, at annual follow-up, up to 5 years) and analyzed for associations with gene polymorphisms.

### DNA extraction and HLA-G14bp ins/del polymorphism

For each patient, genomic DNA was obtained from peripheral blood using commercial kits (QIAamp DNA blood kit, Qiagen, Hilden, Germany) and stored frozen at − 20 °C until use. DNA concentration and purity were spectrophotometrically assessed.

The HLA-G14bp ins/del polymorphism in exon 8 was detected as previously described [19]. In brief, genomic DNA (100 ng) was amplified in a 25 μl reaction, using specific primers; polymerase chain reaction (PCR) products, 210 bp (del) and/or 224 bp (ins), were visualized by electrophoresis on a 4% agarose gel (NuSieve GTG agarose) containing ethidium bromide.

### IL-10, TGF-β, IL-6, TNF-α, and IL-18 gene polymorphisms

Cytokine gene polymorphisms were detected on genomic DNA. IL-10(−1082G > A), (− 819 T > C) and (−592A > C) (promoter region, haplotypes GCC, ACC and ATA), TGF-β(+ 869 T > C) and (+915C > G), (codons 10 and 25, exon 1), IL-6(−174G > C) and TNF-α(−308G > A) genotypes were assessed using a polymerase chain reaction-sequence-specific primers (PCR-SSP) method, according to manufacturer instructions (Cytokine genotyping Primer Pack, Pcytgen, One Lambda, Inc., CA, USA). In each PCR, internal control was included; PCR products were visualized on a 2% agarose gel electrophoresis containing ethidium bromide (0.5 mg/ml). A DNA ladder was present in each gel, and the molecular weight of the PCR products were checked. Allele, genotype, and haplotype frequencies were reported; based on previous data on functional evidence, genotypes, and haplotype combinations were also defined as high, intermediate and low cytokine producers [20].

IL-18(−137G > C) (rs187238) and (−607C > A) (rs1946518) were genotyped using Taqman allelic discrimination assays (Applied Biosystems, CA, USA) on a StepOne Real-Time PCR system.

An Italian population sample of randomly-selected unrelated donors was used as a reference for comparisons of allele/genotype/haplotype frequencies.

### Statistical analysis

Allele, genotype, and haplotype frequencies were calculated. Chi-squared test was used for the analysis of associations between gene polymorphisms and clinical variables of post-transplant risk. Odds ratio (OR) and its 95% confidence interval (CI) were estimated. Comparisons between groups and correlation between variables were examined by parametric (t-test/paired t test, Pearson’s correlation) and non-parametric tests (Mann Whitney U test/Wilcoxon, Spearman’s correlation coefficients), as appropriate. To explore genotype effects and interactions, changes in clinical markers at each time point (pre-transplant and 1 to 5 years after transplant, within-subject factor) between genotypes (between-subject factor) were analyzed using an ANOVA for repeated measures; adjustment for potential confounders was performed. A Bonferroni post hoc test was used to assess the significance level for differences between the individual time points.

Analyses were conducted using an SPSS statistical package (SPSS Inc., Chicago, IL) and Arlequin software [21]. Two-tailed *p* values < 0.05 were considered statistically significant. All tests were two-sided.

## Results

### Patients

Clinical features of transplanted patients (*n* = 173) are reported in Table 1.

All patients were not pre-emptive and not re-transplanted recipients with up to a five-year follow up recorded data, except three patients who had graft loss after a three-year follow-up. Mean age at transplant was 46.77 ± 10.60 years.

**Fig. 1:**
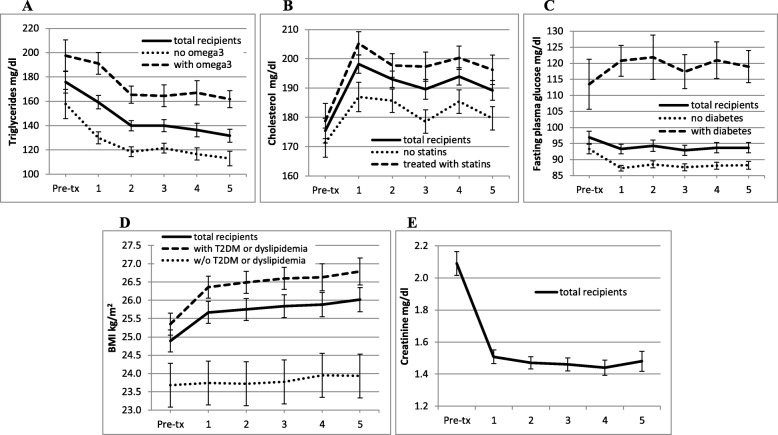
Variations of pre-post transplant triglycerides, total cholesterol, fasting plasma glucose, BMI and blood creatinine. **a** blood triglycerides in recipients with or w/o post-transplant treatment with omega-3 fatty acids (total patients, 2 yrs., *p* = 0.001; 3 yrs., *p* = 0.005; 4 yrs., p = 0.005, 5 yrs., p = 0.001, compared with pre-transplant triglycerides); **b** total cholesterol in recipients with or w/o post-transplant treatment with statins (total patients, 1 yr, *p* < 0.001, 2 yrs., *p* = 0.003, 3 yrs., *p* = 0.022, 4 yrs., *p* = 0.03, compared with pre-transplant cholesterol); **c** fasting plasma glucose in patients with or w/o diabetes (pre-transplant or NODAT); **d** BMI in recipients with or w/o dyslipidemia or T2DM (total patients, 1-5 yrs., p < 0.001, compared with pre-transplant BMI); **e** creatinine (1-5 yrs., p < 0.001, compared with pre-transplant creatinine)

Variations of pre/post- transplant blood triglycerides, total cholesterol, fasting plasma glucose, BMI and creatinine are reported in Fig. 1, where the trend for total patients and subsets treated for dyslipidemia or diabetes is reported. Total cholesterol significantly increased after 1 year (total patients, pre-transplant, 174.07 ± 41.48 mg/dl; after transplant, 1 year: 199.16 ± 38.69 mg/dl, *p* < 0.001), while triglycerides and creatinine were significantly reduced after transplantation (total patients, triglycerides, from 175.90 ± 91.31 mg/dl pre-transplant to 139.95 ± 59.65 mg/dl at 3 years and 131.68 ± 53.63 mg/dl at 5 years post-transplant, *p* < 0.01; creatinine: from 2.10 ± 0.96 mg/dl pre-transplant to 1.46 ± 0.49 at 3 years and 1.48 ± 0.64 mg/dl at 5 years post-transplant, *p* < 0.001). BMI significantly increased over time after transplantation (total patients, from 24.75 ± 4.02 kg/m^2^ pre-transplant to 25.75 ± 4.00 kg/m^2^ 3 years and 26.02 ± 4.26 kg/m^2^ 5 years post-transplant, *p* < 0.005). In particular, post hoc test with Bonferroni correction showed a significantly increased BMI from pre- to post-transplant (1–5 years, *p* < 0.005) and between one-two years and five years assessments after transplantation (*p* = 0.016 and *p* = 0.043, respectively), as compared with pre-transplant BMI. Post-transplant BMI was correlated with age at transplant (*p* < 0.006), pre-transplant BMI (*p* < 0.001) and creatinine (*p* < 0.005).

### Obesity, dyslipidemia, diabetes mellitus

Based on BMI values, patients were grouped into normal range weight (BMI < 25 kg/m^2^), overweight (BMI 25–29.99 kg/m^2^) and obese (BMI ≥ 30 kg/m^2^), according to World Health Organization (WHO) criteria [22].

Prevalence of obesity ranged from 11% (19/173) pre-transplant to 13% (23/173) at 1 year post-transplant, and 17% (29/170) at 5 years post-transplant**,** without significant differences between men and women.

**Fig. 2:**
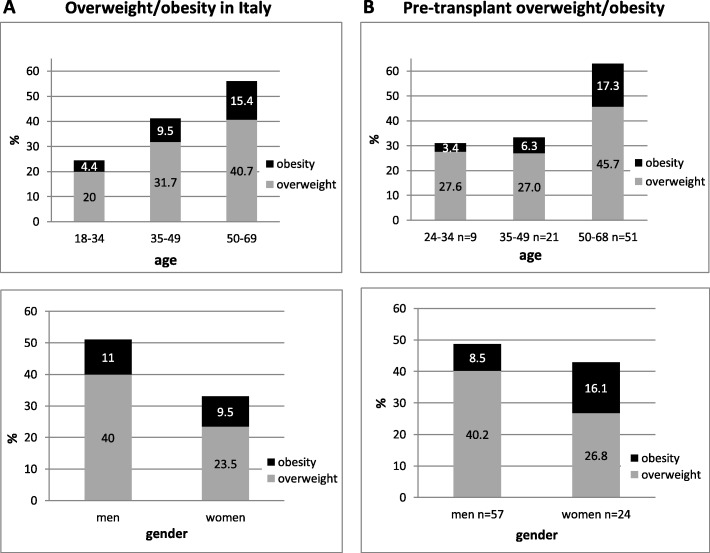
**a** Overweight/obesity in Italian population [23]; **b** Overweight/obesity in pre-transplant recipients (present study)

Data of pre-transplant overweight/obesity were consistent with those of the Italian population (obesity, 10.2%, overweight 31.7%), [23] with slight differences in age and gender distribution (Fig. 2).

In pre-transplant assessment, eight patients were classified underweight according to WHO classification (BMI < 18,5); two of these patients maintained mild thinness during the post-transplant monitoring, while the remaining patients entered the normal weight group during the post-transplant period.

Seventy-two percent of patients (124/173) were treated for post-transplant dyslipidemia: 60% patients (104/173) were under treatment with statins (hypercholesterolemia) and 48% (83/173) with omega**-**3 fatty acids (hypertriglyceridemia). Patients treated for post-transplant dyslipidemia, as compared with non-treated patients, had significantly higher age at transplant (48.71 ± 10.36 vs. 41.47 ± 9.46 years, *p* < 0.001), fasting plasma glucose (pre-transplant: 99.73 ± 26.85 vs. 89.48 ± 16.77 mg/dL, *p* = 0.002; after transplant, 3 yrs.: 95.40 ± 22.12 vs. 86.24 ± 13.82 mg/dL, *p* = 0.017) and BMI (pre-transplant: 25.32 ± 4.03 vs. 23.72 ± 4.02 kg/m^2^, *p* = 0.037; after transplant, 3 yrs.: 26.58 ± 3.98 vs. 23.88 ± 3.67 kg/m^2^, p < 0.001).

In 5.2% of patients (9/173), type 1 diabetes mellitus (T1DM) was diagnosed before transplantation or was responsible for renal failure; 12.7% (22/173) had new-onset diabetes after transplantation (NODAT).

### Gene polymorphisms

Distribution of HLA-G14bp ins/del genotypes and alleles in transplant recipients is reported in Table 2 and compared with those of the Italian population and of other studies in Caucasians**.**

**Table 2:** HLA-G14bp genotypes/alleles in kidney transplant recipients and comparison between populations

	***Present study***		***Italian population***				***Brasilian population***	
	Recipients	Controls^a^	Controls^b^	Controls^c^	Recipients (Sardinia)^d^	Controls (Sardinia)^d^	Recipients^e^	Controls^e^
	*(n = 173)*	*(n = 118)*	*(n = 400)*	*(n = 102)*	*(n = 418)*	*(n = 371)*	*(n = 83)*	*(n = 97)*
***Genotypes***	* n (%)*	* n (%)*	* n (%)*	* n (%)*	* n (%)*	* n (%)*	* n (%)*	* n (%)*
−14/−14, 210 bp	**42 (24.3)**	40 (33.9)	124 (31.0)	36 (35.3)	**139 (33.5)**	119 (32.2)	**21 (25.3)**	32 (33)
-14/+ 14, 210/224 bp	**93 (53.7)**	62 (52.5)	216 (54.0)	47 (46.1)	**198 (47.4)**	178 (47.9)	**37 (44.6)**	49 (51)
+ 14/+ 14, 224 bp	**38 (22.0)**	16 (13.6)	60 (15.0)	19 (18.6)	**81 (19.4)**	74 (19.9)	**25 (30.1)**	16 (16)
Alleles								
-14, 210 bp	**177 (51.2)**	142 (60.2)	464 (58.0)	119 (58.3)	**476 (57.0)**	416 (56.1)	**79 (47.6)**	113 (58.2)
+ 14, 224 bp	**169 (48.8)**	94 (39.8)	336 (42.0)	85 (41.7)	**360 (43.0)**	326 (43.9)	**87 (52.4)**	81 (41.8)

References: ^a^unrelated donors, reference Italian population; ^b^Fabris A., 2011 [24];^c^Sizzano F., 2012 [25]; ^d^Littera R [14].; ^e^Crispim J.C., 2008 [13]

As regards the assayed gene polymorphisms, no significant differences were observed between transplanted patients and controls (Supporting information Figure 6) and between genotypes according to characteristics of kidney transplant recipients described in Table 1 (gender, age at transplant, dialysis mode, time on dialysis, immunosuppressive therapy, cold ischemia time, acute rejection and organ loss, primary nephropathy).

### HLA-G14bp ins/del polymorphism and post-transplant risk factors

A comparative analysis to evaluate the possible effect of genotypes on some of the most important clinical and biochemical parameters of recipients was performed.

HLA-G14bp ins/ins genotype frequencies were associated with presence of obesity at pre-transplant (*p* = 0.008, OR 3.88, CI 1.45–10.41) and at 3–5 years after transplant (*p* = 0.002, OR 4.48, CI 1.76–11.41, *p *= 0.002, OR 3.95, CI 1.67–9.34 and *p *= 0.028, OR 2.68, CI 1.13–6.39, respectively).

**Fig. 3:**
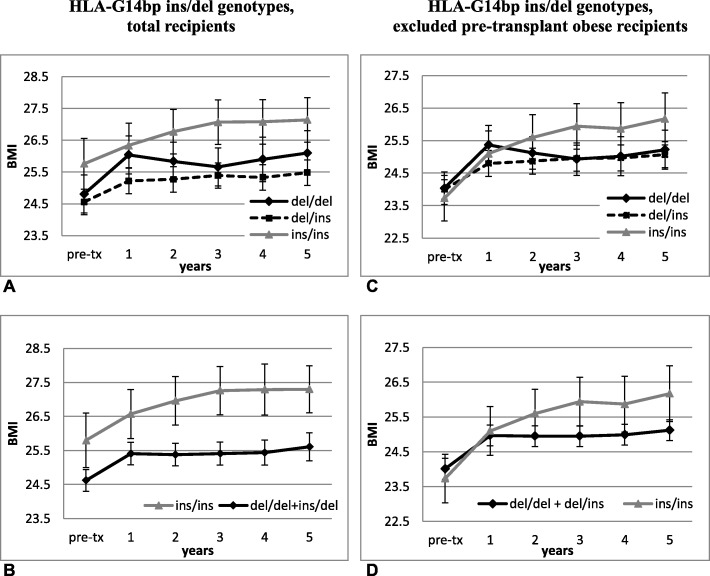
Pre/post-transplant measurements of BMI (kg/m^2^) and HLA-G14bp ins/del genotypes in kidney transplant recipients: **a** and **b**: total recipients; **b** recessive model: significantly increased BMI in HLA-G14bp ins/ins, as compared with ins/del + del/del recipients, at 3 and 4 years (*p* = 0.033 and 0.044, respectively); using repeated-measures ANOVA, it was observed a significant main effect of HLA-G14bp genotypes on BMI (*p* = 0.049), with no significant interaction between BMI and HLA-G14bp genotypes; **c** and **d**: pre-post transplant BMI when pre-transplant obese patients (BMI > 30) were excluded from the analysis (*n* = 154); **d** recessive model: significant pre-post transplant weight gain in both ins/ins and ins/del + del/del carriers (*p* < 0.005) and significant interaction between BMI and HLA-G14bp genotypes (F = 4.45, *p* = 0.007); after transplantation, only carriers of ins/ins genotype had significantly increased BMI between 1 to 2 (*p* = 0.01), 3 (p = 0.001), 4 (*p* = 0.008) and 5 (p < 0.001) years after transplant. Pre-tx = pre-transplant

**Fig. 4:**
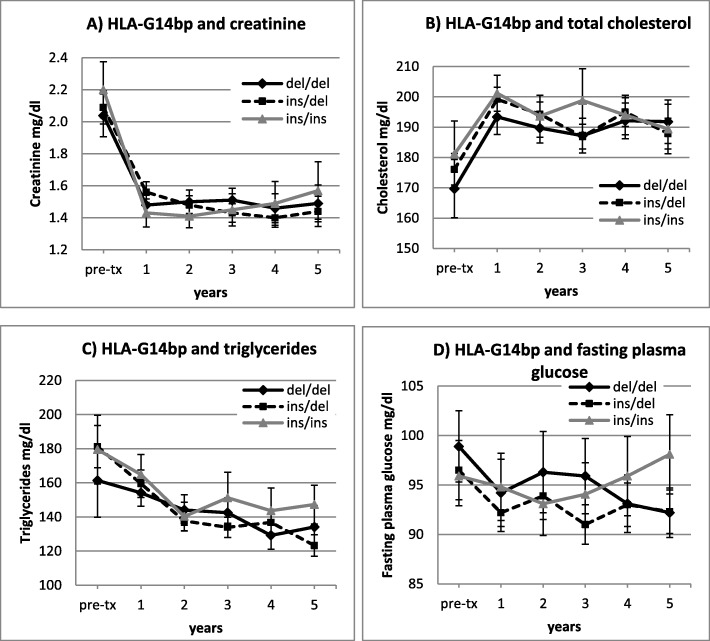
HLA-G14bp genotypes and pre/post-transplant variation of **a**) creatinine, **b**) total cholesterol, **c**) triglycerides and **d**) fasting plasma glucose in kidney transplant recipients (p = ns)

Patients with HLA-G14bp ins/ins genotype had higher BMI after transplantation (Fig. 3): differences were statistically significant at 3 and 4 yrs., (*p* = 0.033 and 0.044, respectively), while BMI before the transplant was not significantly different among HLA-G14bp genotypes (Fig. 3b). No correlation was found between HLA-G14bp ins/del genotypes and blood cholesterol, triglycerides, fasting plasma glucose, creatinine (Fig. 4).

By using a GLM analysis (repeated-measures ANOVA), a slight but significant effect of HLA-G14bp genotypes on BMI was observed (recessive model, ins/ins vs. ins/del + del/del, F = 3.95, *p* = 0.049 (Fig. 3b); no significant interaction between BMI and HLA-G14bp genotypes was detected, which means that BMI changed in time, but in the same way in the three groups based on HLA-G14bp genotypes.

Excluding pre-transplant obese recipients (BMI > 30) from the analysis, it was possible to better evaluate effects of transplantation on weight gain: a significant interaction between BMI and HLA-G14bp genotypes was evidenced (F = 4.45, *p* = 0.007); paired t-tests showed a substantial increase of BMI between one and the next years (*p* < 0.01) only in carriers of the ins/ins genotype, while carriers of other genotypes did not show further significant weight gain after the initial post-transplant increase (Fig. 3d). Differences between groups at each genotype are reported in Fig. 3.

**Fig. 5:**
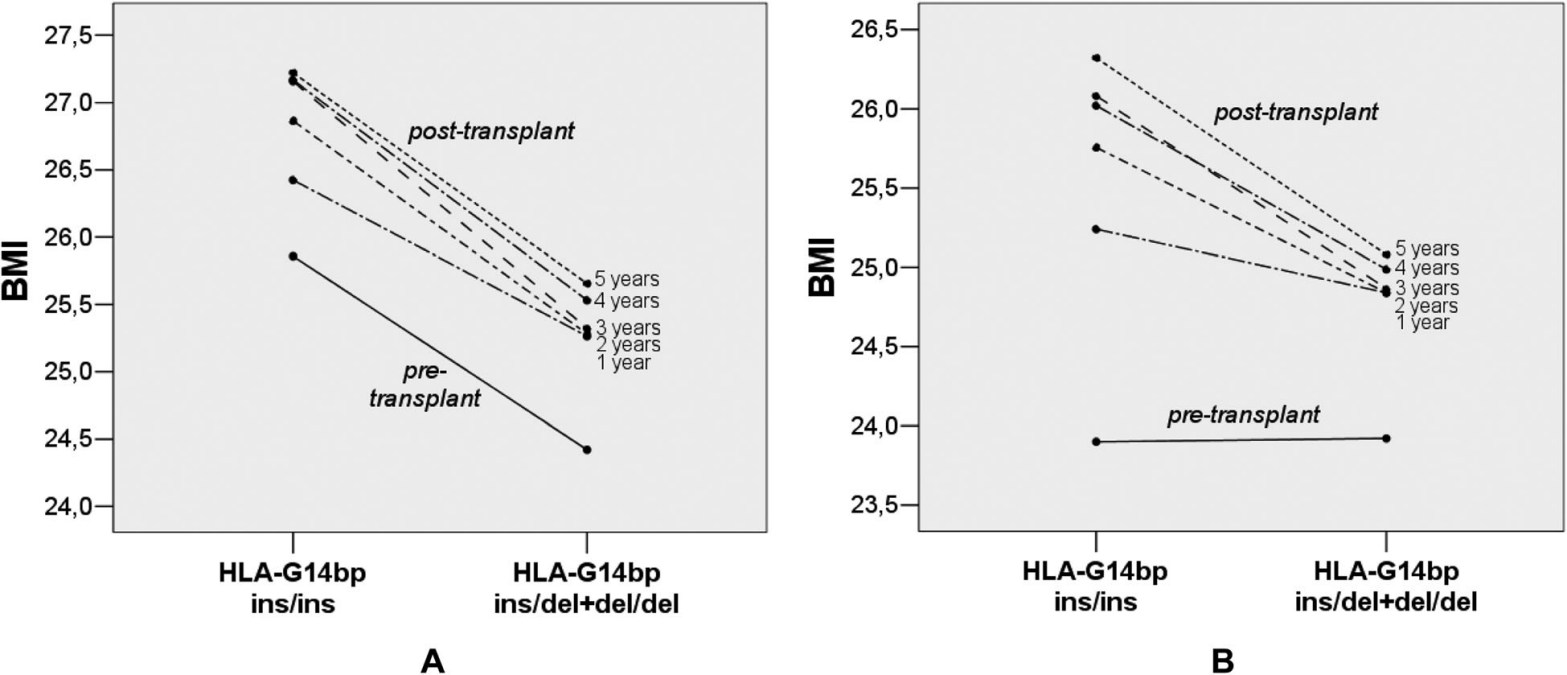
Estimated marginal means of BMI and HLA-G14bp genotypes (recessive model), with gender and age at transplant as covariates; **a** total recipients: effect of HLA-G14bp ins/ins genotype (*p* = 0.029) and age at transplant (*p* = 0.001) on BMI (pre-transplant, 1 to 5 years after transplant), with no interaction between HLA-G14bp genotypes and BMI over time (BMI was significantly increased between pre- to post-transplant in both ins/ins and ins/del + del/del carriers); **b** when pre-transplant obese recipients were excluded from the analysis, a significant interaction between HLA-G14bp genotypes and BMI over time was found (*p* = 0.009)

These results were confirmed after adjustment for the effect of gender and age at transplant: a slight but significant effect of HLA-G14bp genotypes (F = 4.87, *p* = 0.029) and age at transplant (F = 12.00, *p* = 0.001) on BMI was observed, with no interaction between HLA-G14bp genotypes and BMI over time **(**Fig. 5a). When pre-transplant obese patients were excluded from the analysis, the significant interaction between HLA-G14bp and BMI was confirmed (recessive model, F = 4.26, *p* = 0.009) (Fig. 5b).

Statistical significance was maintained when recipients with diabetes (NODAT, *n* = 22, or pre-transplant, *n* = 7) were excluded from the analyses (data not shown).

### Cytokine gene polymorphisms and post-transplant risk factors

IL-10(−1082G > A),(− 819 T > C), (−592A > C), TGF-β(+ 869 T > C), (+915C > G) IL-6(−174G > C), TNF-α(−308G > A) and IL-18(−137G > C),(−607C > A) cytokine polymorphisms were not correlated with the examined factors (BMI/obesity, blood cholesterol, triglycerides, fasting plasma glucose, creatinine) and their variations along the considered period after transplantation (Supporting information Figure 7: cytokine polymorphism and BMI; other variables: not shown).

As IL-10 and TGF-β genotypes/haplotypes were previously associated with their release in vivo, analysis of results also included phenotypes, indicated as “*high*” (=*high producers*), “*intermediate*” (=*intermediate producers*) and “*low*” (=*low producers*), as previously reported [20]. Combinations of genotypes did not demonstrate significant effects on the evaluated parameters. The significantly increased frequency of IL-10-1082G/G genotype in HLA-G14bp ins/ins positive individuals, previously reported in healthy controls [17], was not confirmed in the present study. Neither effect of gender nor correlation with age at transplant were observed through genotypes.

## Discussion

The research of genetic markers predictive of post-transplant complications and long-term graft survival is a frequent target in transplantation.

Here we explored the possible association of some functional polymorphisms of molecules involved in both tolerance and low grade inflammation with some parameters of increased risk for long-term complications in a group of kidney transplant recipients, as some cytokines and non classical HLA molecules involved in immunological mechanisms of allograft rejection could also interfere with cardiovascular and metabolic risk.

Monitoring some of the most common parameters up to 5 years post-transplant, an increase of BMI/obesity, fasting plasma glucose and cholesterol was registered, as expected, while both creatinine and triglycerides reduce over time. When these parameters were evaluated in association with cytokine genotypes and alleles, no predictive effects were found. Results showed a correlation of HLA-G14bp genotypes with BMI, which significantly increased in subsets of recipients carrying the ins/ins genotype. Considering overall patients, BMI changes in time, in the same way in the three groups based on HLA-G14bp, while a significant effect of HLA-G14bp ins/ins genotype on post-transplant weight gain was detected. Patients with HLA-G14bp ins/ins were more likely to develop obesity at 3 years after transplantation. No correlations were found with other parameters (blood cholesterol, triglycerides, fasting plasma glucose, creatinine). No significant associations of gene polymorphisms with post-transplant diabetes or rejection were found in this study, hence data on calcineurin inhibitors levels and specific anti rejection treatments have not been reported at this time. Effects of HLA-G14bp genotypes on post-transplant BMI were confirmed when considering only non-obese transplant candidates in the analysis, after controlling for age at transplant and gender. Effects of HLA-G14bp ins/ins genotype on BMI, although limited, were significant and seemed independent of the presence of diabetes, metabolic syndrome and other risk factors associated with BMI increase and low-grade inflammation. Immunosuppressive regimens (tacrolimus or cyclosporine) did not affect this result.

Cytokine gene polymorphisms were not correlated with the examined variables and their variations during the considered period after transplantation. Neither effect of gender nor correlation with age at transplant was observed.

From the results of this study, HLA-G14bp ins/del polymorphism, previously involved in transplantation for its tolerogenic activity, seems to have a potential role in the development of obesity. This observation, carried out in transplant recipients, highlighted this potential link between HLA-G and obesity which, if confirmed (especially in long term evaluations), could also be of interest to other clinical contexts. Results of pre-transplant detections would not exclude an effect of HLA-G14bp polymorphism on obesity and inflammation associated with end stage renal disease. HLA-G is considered an anti-inflammatory factor that modulates immune cell responses [26]. Obesity and T2DM are characterized by low-grade inflammation [27], and a relationship seems to exist between HLA-G, obesity, and T2DM: in a study of HLA-G production in T2DM, sHLA-G was associated with higher BMI, cholesterol and blood pressure.

Recently, higher concentrations of sHLA-G have been observed in overweight-obese women during pregnancies, compared with healthy controls and with preeclamptic overweight/obese pregnancies [28]. Crosstalk between adipocytes, which could act as antigen presenting cells (APC), T cells and regulation of adipose tissue inflammation has been proposed and may involve cytokines and HLA-G expression [29].

From literature reviews on obesity-related genes, the role of pro-inflammatory cytokines (TNF-α, IL-6, IL-1) is known, while that of anti-inflammatory molecules is less studied. Cytokine gene polymorphisms were also associated with obesity, metabolic syndrome, and cardiovascular risk [8–10, 30–41]. Nevertheless, we found no significant associations with cytokine gene polymorphism. In addition, the previously observed association of HLA-G14bp and IL-10-1082G/G (the *high producer* IL-10-1082 genotype, most frequently found in individuals carrying the HLA-G14bp ins/ins [17]), was not present in transplanted patients.

Immunosuppressive protocols have changed over the years, with the replacement of azathioprine by mycophenolate mofetil (MMF) and the introduction of tacrolimus, monoclonal antibodies and mTORi. Therefore, the high-level immunosuppression achieved in the last years has made potential effects of gene polymorphisms less detectable, although they could affect the onset of complications, quality of life and overall graft survival in the long term. With these premises, what we could expect were small effects of genotypes that, in this particular condition, could highlight novel pathogenic mechanisms.

In the future, a deeper knowledge of diet-gene interaction [42] could produce novel genetically driven approaches in subsets of well-selected patients. Obesity is strongly affected by genetic components that, indeed, in physiological conditions, could be effectively counteracted by lifestyle and environmental interactions. Transplantation creates a particular condition in which a genetic substratum could act in synergy with external predisposing factors such as immunosuppressive therapy and inflammation. In this situation, it becomes particularly relevant to discover possible predisposing conditions. It should be considered that the effects of new biologic drugs can be modified by genetic polymorphisms. For example, the fusion protein cytotoxic T-lymphocyte antigen (CTLA)4-Ig, introduced for prevention of rejection, mainly exerts its tolerogenic function through sHLA-G release [43].

## Conclusions

We analyzed associations of some gene polymorphisms with pre/post-transplant variations of the main risk factors for metabolic/cardiovascular diseases, like excess body weight, increased blood lipids and fasting plasma glucose, and we found out a potential relationship between post-transplant weight gain and HLA-G14bpins gene variant in kidney transplant recipients. The particular condition of newly transplanted patients (the start of immunosuppressive therapy and a careful post-transplant monitoring that includes metabolic parameters affected by this treatment) have allowed to uncover this potentially interesting association. This novel association could add new elements to the study of obesity susceptibility factors and to the knowledge of the role and functions of HLA-G molecules in diseases and transplantation.

## Supplementary information

**Additional file 1.** Supporting information - Figure6 Cytokine genotypes, alleles and haplotypes in kidney transplant recipients and controls.

**Additional file 2.** Supporting information - Figure7 Cytokine genotypes and pre/post-transplant BMI in kidney transplant recipients.

## Data Availability

The data that support the findings of this study are available within the article and its supplementary information files or from the corresponding author on reasonable request.

## Abbreviations

APC: Antigen presenting cells
BMI: Body mass index
bp: Base pair
CD25: Cluster of differentiation 25
CI: Confidence interval
CTLA-4: Cytotoxic T-Lymphocyte Antigen 4
del: Deletion
DNA: Deoxyribonucleic acid
GLM: General linear model
HLA: Human leukocyte antigen
IL: Interleukin
ins: Insertion
LPS: Lipopolysaccharides
MMF: Mycophenolate mofetil
mRNA: Messenger ribonucleic acid
mTORi: Mammalian target of rapamycin inhibitors
NODAT: New onset diabetes after transplantation
OR: Odds ratio
PBMC: Peripheral blood mononuclear cells
PCR: Polymerase chain reaction
SSP: Sequence specific primers
T1DM: Type 1 diabetes mellitus
T2DM: Type 2 diabetes mellitus
TGF: Transforming growth factor
TNF: Tumor necrosis factor
WHO: World health organization
