# Supplementary material for: HLA-G14bp ins/del polymorphism and post-transplant weight gain in kidney transplantation: potential implications beyond tolerance
Source: BMC Nephrol. 2020 Mar 30;21:109. doi: 10.1186/s12882-020-01752-6 (PMC7104538; doi:10.1186/s12882-020-01752-6)
Supplement: Supplementary file 1 — Additional file 1. Supporting information - Figure6 Cytokine genotypes, alleles and haplotypes in kidney transplant recipients and controls. [file 12882_2020_1752_MOESM1_ESM.docx]

**Supporting information - Figure 6** - Cytokine genotypes, alleles and haplotypes in kidney transplant recipients and controls. ^a^IL-10-592A>C polymorphism has the same frequencies of IL-10-819T>C.
