# Supplementary material for: HLA-G14bp ins/del polymorphism and post-transplant weight gain in kidney transplantation: potential implications beyond tolerance
Source: BMC Nephrol. 2020 Mar 30;21:109. doi: 10.1186/s12882-020-01752-6 (PMC7104538; doi:10.1186/s12882-020-01752-6)
Supplement: Supplementary file 2 — Additional file 2. Supporting information - Figure7 Cytokine genotypes and pre/post-transplant BMI in kidney transplant recipients. [file 12882_2020_1752_MOESM2_ESM.docx]

**Supporting information - Figure 7** - Cytokine genotypes and pre/post-transplant BMI in kidney transplant recipients. No significant variations were evidenced. Data are expressed as mean ±SE. Pre-tx: pre-transplant BMI; 1-5: years after transplantation.
